# The diode laser as coadyuvant therapy in the non-surgical conventional treatment of peri-implant mucositis: A systematic review and meta-analysis

**DOI:** 10.4317/jced.57630

**Published:** 2020-12-01

**Authors:** Rebeca Sánchez-Martos, Andrea Samman, Mattia Priami, Santiago Arias-Herrera

**Affiliations:** 1 Universidad Europea de Valencia. Faculty of Health Sciences. Department of Dentistry

## Abstract

**Background:**

The present study systematically reviewed randomized controlled trials (RCT) to investigate the effect of diode laser therapy in the management of peri-implant mucositis.

**Material and Methods:**

The electronic databases were searched until January 2020. Outcome measures were bleeding on probing (BOP), plaque index (PI) and probing depth (PD). The addressed PICO question was: Is the diode laser therapy effective reducing the signs of inflammation as an adjunctive element in the non-surgical treatment of peri-implant mucositis?.

**Results:**

Eight randomized clinical trials (RCTs) were included in the systematic review for qualitative synthesis and three in the meta-analysis for quantitative synthesis. All studies included in the quantitative synthesis have low risk of bias according to the Cochrane collaborations’ tool. Diode laser as coadyuvant therapy significantly reduced plaque index (SMD: -1.24; -0.47/-1.53) but not in bleeding on probing (SMD: -0.84; -0.31/-1.53) or probing pocket depth (SMD: -1.36; -0.28/-1.69). Non-statistically significant reductions in peri-implant bleeding on probing and in probing pocket depth were also observed in the test groups of most studies included in the meta-analysis.

**Conclusions:**

The results should be interpreted cautiously due to the great heterogeneity in the methodology of the studies included in the systematic review. However the meta-analysis suggests that the use of diode lasers, as an adjunct in conventional non-surgical treatment of peri-implant mucositis, is promising in reducing the clinical signs of peri-implant mucositis, especially reducing the perii-implant plaque index.

** Key words:**Peri-implant diseases, peri-implant mucositis, laser therapy, diode laser.

## Introduction

Nowadays, the treatment of dental absences with dental implants has predicTable results with high success rates and long-term stability in resolving cases of partial and total edentulism (1). However, the clinical stability of long-term implant treatment is compromised by peri-implant pathologies which are present with a high incidence rate (2).

The definition of peri-implant mucositis has evolved over the years. At the 6th European Workshop of Periodontology in 2008, peri-implant mucositis was described as an inflammatory lesion that resides in the mucosa, that can be identified clinically by redness and swelling of the soft tissue, but bleeding on probing is currently recognized as the important feature (3). This description was considered adequate also at the 7th European Workshop of Periodontology in 2011 (4). The 8 th European Workshop on Periodontology in 2012 (5) agreed that the definition established in the previous European Workshops published in 2008 (3) and 2011 (4) should be adopted to define peri-implant mucositis and clarifies that clinical signs, such as suppuration and increases in the basal probing pocket depth could appear in the pathology (5). According to the latest definition provided by the “Consensus report from 2017 World Workshop on Periodontology”, peri-implant mucositis is defined as an inflammatory disease of the mucosa surrounding an endosseous implant without loss of supporting peri-implant bone. The clinical sign of the inflammation is bleeding on probing, while additional signs can include erythema, swelling and suppuration (6).

Peri-implant pathologies have an increasing prevalence. In Spain, the last review carried out by Rodrigo D *et al.* in 2018 showed a prevalence of 51% for peri-implant pathologies, with a mucositis rate of 27% (7). Internationally, the most recent review carried out by Lee *et al.* in 2017, showed a subject-based prevalence of 19.83% for peri-implantitis and 46.83% for peri-implant mucositis (8), similar to the results carried out by Derks & Tomasi in 2015 (2) and Atieh *et al.* in 2013 (9).

The major etiological factor for developing peri-implant mucositis is biofilm accumulation (10,11). Smoking, lack of compliance with supportive implant therapy, radiation, diabetes, therapy shape of the restoration, dimension of keratinized tissue mucosa, excess of cement are been documented as factor risk of peri-implant mucositis (12,13). The treatment protocols for peri-implant pathologies are empirically based on the protocols for the treatment of periodontal infections (14). Thus, surface debridement represent the basic element for treatment of peri-implant pathologies (12). However, this conventional treatment has limitations in the resolution of peri-implant mucositis (15,16). In recent years, new techniques have been developed such as the use of lasers that, according to recent articles, have the potential to enhance the results of conventional treatment of peri-implant diseases, presenting a valid option, as an adjunct to conventional treatment (17).

At present there are very few studies that evaluate the effect of the diode laser as an adjunctive treatment in the non-surgical treatment of peri-implant mucositis. In addition, the heterogeneity of the studies induces conflicting results in the studies. It is necessary, therefore, to conduct a systematic review and meta-analysis of published trials for the purpose of synthesizing, and to clarify the published literature. Thus, this systematic review and meta-analysis aimed to evaluate the additional effect of diode laser therapy in the nonsurgical management of peri-implant mucositis 

## Material and Methods

-Protocol and focused question

The review was registered in PROSPERO, an International Prospective Register of Systematic Reviews under registration number: CRD42020183294. The Preferred Reporting Items for Systematic Review and Meta- Analysis (PRISMA) guidelines was followed to perform this systematic review (18). The following focus question was employed according to the population, intervention, comparison, and outcome study design. Is the diode laser therapy effective reducing the signs of inflammation as an adjunctive element in the non-surgical treatment of peri-implant mucositis?.

-Selection criteria 

All studies selected for the systematic review had to follow the following inclusion criteria. Regarding the type of study, they had to be randomized controlled clinical studies (RCT) or cohort; the sample should be made up of adult subjects diagnosed with peri-implant mucositis. The experimental group should have been assigned to non-surgical periodontal treatment with complementary diode laser therapy compared to the control group that should have received non-surgical treatment of peri-implant mucositis. The studies also had to have a minimum follow-up of 3 months and take into account at least the variable of bleeding on probing. Animal studies and articles published before 2010, not available in Spanish or English, were excluded; Case reports, case series, pilot studies, narrative literature reviews, and letters to the editor were also excluded.

-Search strategy 

The authors performed an initial electronic research in MEDLINE via Pub-Med and Cochrane Central Register of Controlled Trials until January 2020. The literature search was conducted using the combinations of the following Medical Subject Heading (MeSH) and text words: “(peri-implant diseases OR peri-implant mucositis OR peri implant pathology OR peri implant soft tissue alterations OR peri implant soft tissue complications) AND (laser therapy OR diode laser OR photodynamic therapy OR diode soft laser OR diode photobiomodulation OR diode stimulation) AND (non surgical subgingival debridement OR non-surgical peri-implant treatment OR mechanical anti-infective therapy OR mechanical therapy OR non-surgical mechanical debridement OR conventional treatment) AND (gingival index OR bleeding on probing OR peri-implant bleeding OR peri implant health index OR peri implant probing depth OR gingival inflammatory response)”. Manual search of reference list was used to identify additional articles. Additional relevant articles were searched manually from the reference lists of full text in order to not exclude any publication of interest.

-Screening methods and data abstraction 

Two reviewers (RS and AS) in duplicate and independently performed the systematic review search. Once the duplicate had been removed, titles and abstracts of all identified studies were screened for eligibility. During this phase, the articles were excluded because they were published before the established date (2010) or because they did not fit the study topic. The full text of all the studies selected in the first phase was read and the inclusion and exclusion criteria were applied. Any disagreement was resolved with discussion between both reviewers until consensus was reached or through arbitration by a third examiner (S.A). The level of agreement was calculated using the k-score according to the criteria of Landis & Koch (19).

Data was extracted from accepted studies, including the following details. Authors name, year of publication, country, subjects (sample size, mean and age range in years 

and male to female ratio) peri-implant diagnostic criteria, study groups, diode laser technical specifications and follow-ups. In addition to the following variables: plaque index (PI), bleeding on probing (BoP) and probing pocket depth (PPD). Plaque index change (ΔPI) Bleeding on probing change (ΔBOP) and probing pocket depth change (ΔPPD) were also calculated.

-Risk of bias in individual studies 

The risk of bias was assessed independently and in duplicate by the two authors  (RS and AS) according to the Cochrane collaborations’ tool (20). Overall, studies were considered as Low Risk of bias if the trial is judged to be at low risk of bias for all domains, Unclear if the trial is judged to raise some concerns in at least one domain for this result and High Risk of bias if the study is judged to be at high risk of bias in at least one domain or the trial is judged to have some concerns for multiple domains. Other sources of bias were also registered and taken into account including: Internal and external validity, statistical analysis, evaluation method, examiner calibration, data reproduction, validation of measurements, placebo and patient compliance.

- Case definitions

Peri-implant mucositis: The most recent definition of peri-implant mucositis is included within the New Classification of Periodontal and Peri-Implant Diseases and Conditions, 2018 (6). This definition will be taken as the current definition of peri-implant mucositits in our review.

• Presence of bleeding and/or suppuration on gentle probing with or without increased probing depth compared to previous   examinations and absence of bone loss beyond crestal bone level changes resulting from initial bone remodeling (6).

All the definitions of peri-implant mucositis used by the included studies are found in Table 1.

**Table 1:**
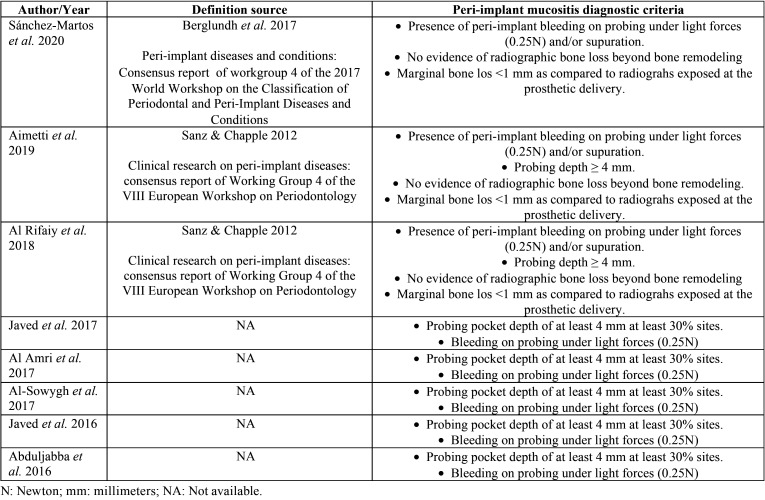
Definition of peri-implant mucositis used in the included studies.

Conventional non-surgical treatment of peri-implant mucositis: Currently there is no gold standard in the treatment of peri-implant mucositis, several protocols have been described over the years based on the experience of treating gingivitis (10).

• The treatment is based on the non-surgical removal of plaque deposits and calculus by using plastic or teflon curettes and establishing good plaque control with proper oral hygiene instructions.

Diode laser therapies: There is no consensus on a gold standard protocol for laser treatment for peri-implant diseases. Two types of diode laser therapy will be considered in this review (21,22).

• Laser therapy (LT): Laser light of specific wavelength, its effect is based on its anti-infective and bio-stimulatory effects Its action is based on its thermal effect.

• Photodynamic therapy (PDT): Laser light of specific wavelength with photosensitizer application.

-Data analysis 

The articles were compared, and the mean values of the primary variables were directly grouped and analysed using standardised mean difference (SMD) and 95% confidence intervals (CI). All analyses were performed with the IBM® SPSS® Statistics version 21.00 software. Statistical significance was defined for a value of *p* <0.05.

## Results

-Study selection

**Figure 1:**
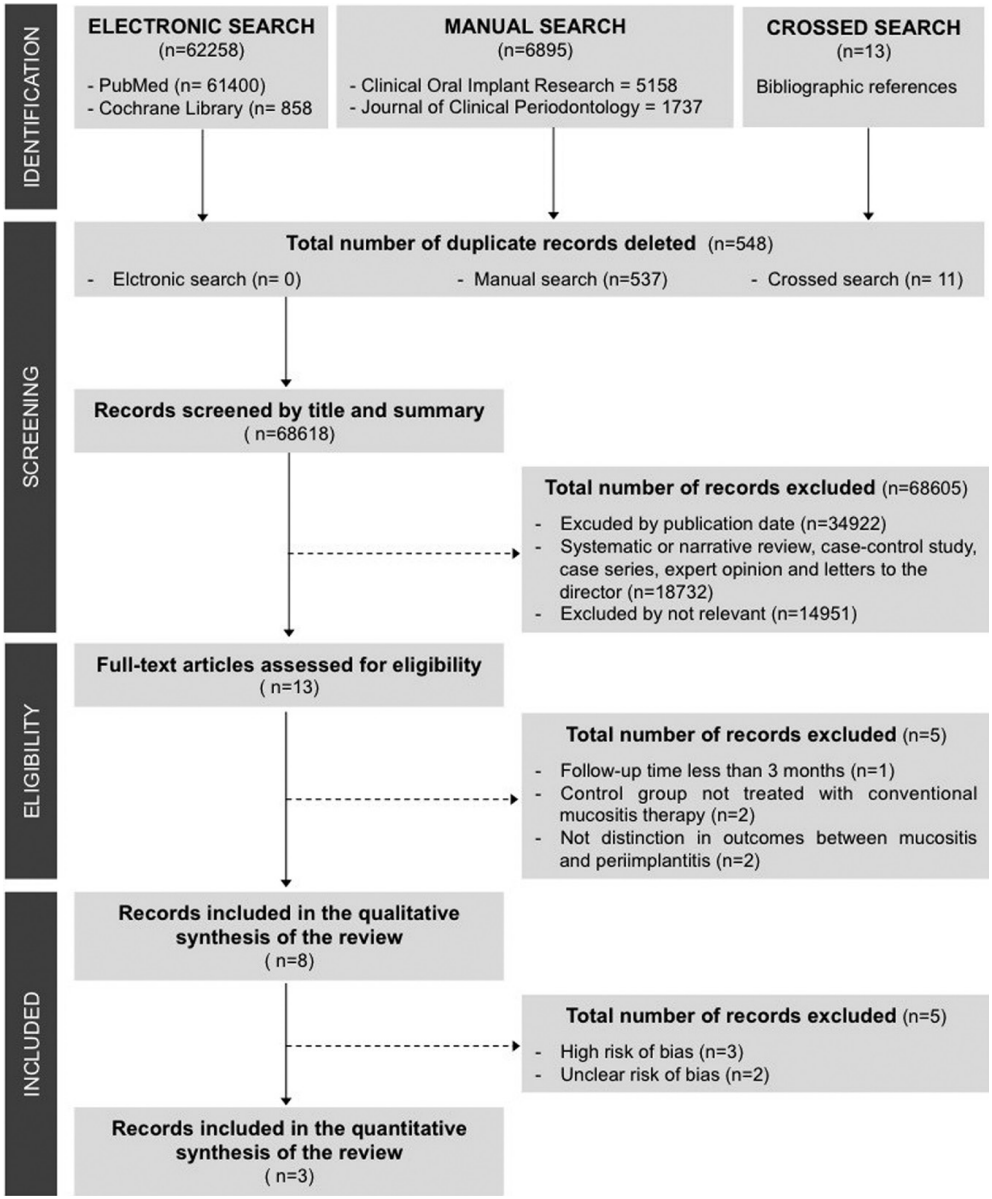
Study selection process and results of the literature search (PRISMA flow diagram).

Searches returned a total of 69166 records. Duplicate papers between sources were removed (n=548). The rest of the studies were screened by title and abstract (n=68618) Of these were eliminated (n=68605), most were excluded by publication date (n=34922), others were not randomized controlled clinical studies (n=18732) and the rest were excluded because they were not relevant for the objective of this review (n=14951). The remaining studies were full text screened (n=13) and five were excluded because they did not meet the inclusion criteria (23-27). All excluded studies are listed in Table 2, which also specifies the reasons for exclusion of each publication. Finally eight studies were included in the qualitative synthesis of the review (17,28-34) and three of these trials were included in the quantitative synthesis of the review (17,28,29). The inter-assessor agreement was excellent at initial screening and full-text eligibility (k = 0.89 and 0.94 respectively) (19). Figure 1 shows flow diagram of study selection process and results of the literature search according to PRISMA guidelines (18).

**Table 2:**
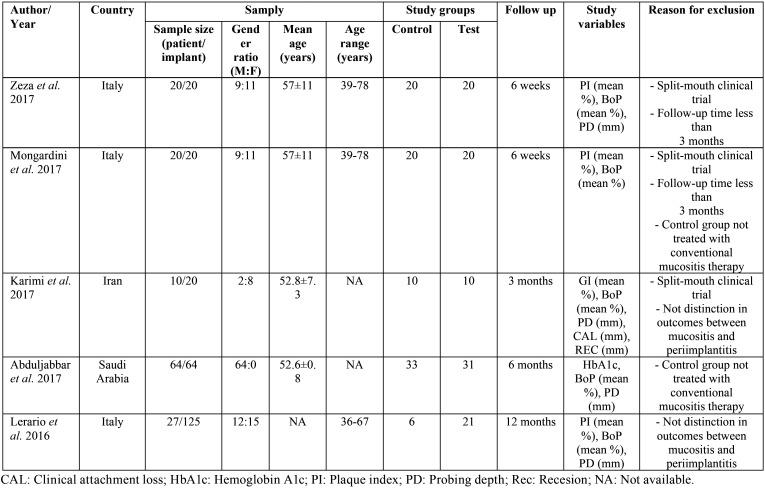
Methodology of excluded studies.

-Characteristics of included studies 

The diagnostic criteria for peri-implant mucositis varied between studies. Four of the eight studies accepted patients who presented dental implants that, when probing with light force (0.25 N), presented bleeding and a depth ≥ 4mm, as well as signs of inflammation in the peri-implant mucosa (30,32-34). Another study established, in addition to the previous parameters, that there had been no variations greater than 1 mm since the bone remodeling phase of the implants (28). The most recent study took into account bleeding after peri-implant sulcus probing after 30 seconds and also that there had been no changes in the level of peri-implant bone beyond bone remodelling (17). One of the publications only established the requirement of presenting gingival inflammation in the peri-implant sulcus (31) and another (29) did not define what criteria was used to diagnose patients with peri-implant mucositis.

The eight selected studies were randomized controlled clinical trials. They were published between 2016 and 2020. The studies were conducted in Spain (17), Italy (28), United States (30,33) and Saudi Arabia (29,31,32,34). A total of 721 patients participated in the studies, 467 men and 254 women, representing 64.7% and 35.2% respectively. The age range of the participants is between 26 and 78 years with an average age of 48.6 years. Regarding the sample of the studies, two publications conducted their trials in healthy patients, specifying the smoking rate by study group (17,28). Other studies used a sample made up only of smokers (30,33) and electronic cigarette smokers (29,32). The remaining two studies conducted their trial in medically compromised patients (31,34), with pre-diabetes and diabetes respectively. The follow-up times of the studies were 3, 6 and 12 months. The most used was the three-month period carried out in five of the eight publications (17,28-30,32). One of the studies followed the sample for 6 months (34) and another two did it for 12 months (31,33). Regarding the variables studied in the trials, all of them studied the changes in bleeding on probing and probing pocket depth. Five of the eight studies also analyzed changes in the plaque index (17,28-30,32). All the analyzed studies used the conventional non-surgical treatment of peri-implant mucositis to treat the control group of their samples. However, only the two most recent studies (17,28) detailed the procedure. All the methodological characteristics of the studies included in the review are summarized in Table 3.

**Table 3:**
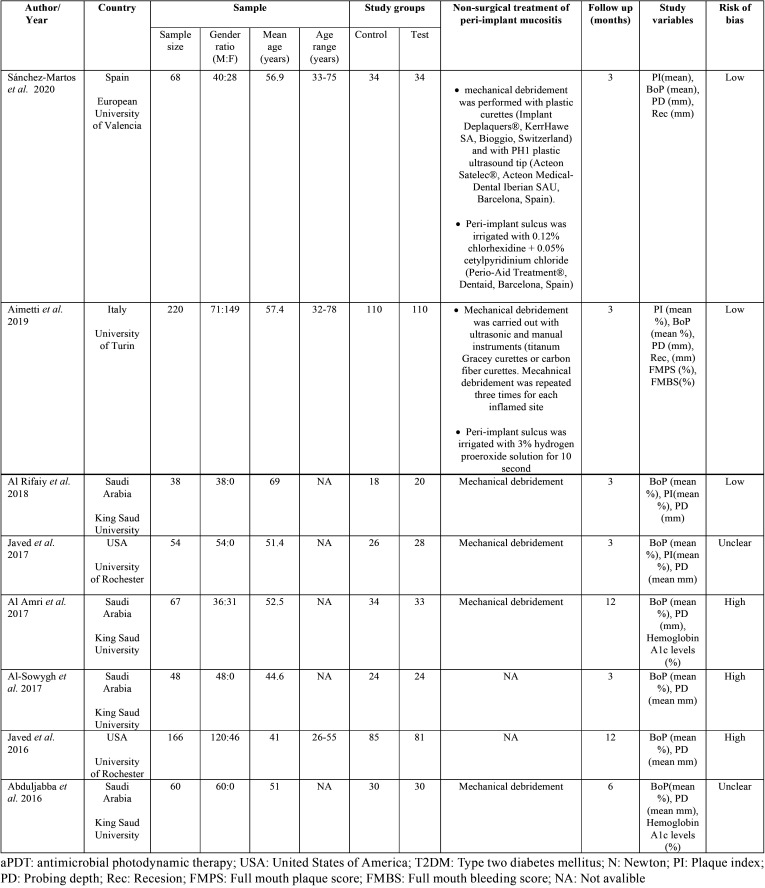
Methodology of included studies.

-Laser and photochemotherapy related parameters 

The technical specifications of the lasers used in the articles are summarized in Table 4. Two of the eight studies analyzed used laser therapy (LT) (17,28), while the rest (29-34) used photodynamic therapy (PDT). These studies (29-31,34) used 0.005% methylthioninium hydrochloride, also known as 3,7-bis phenothiazine-5-ium chloride as photosensitizer. While the other studies that use PDT (32,33) do not specify which element used as a photosensitizer. Studies using LT (17,28) introduced the concept of biostimulation. They applied a pre-irradiation to the patients, using a tip with a larger diameter, 1 cm and 0.7 cm, to achieve a defocused light beam with less power. Both studies applied these beams before the main laser therapy.

**Table 4:**
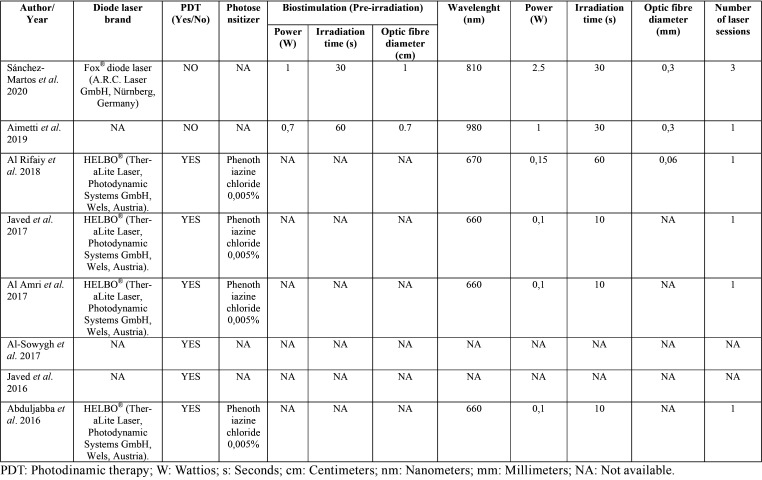
Laser and photosensitizer parameters of included studies.

As for the wavelength used, the highest are found in studies using LT ranging from 810 (17) to 980 nm (28). The rest use lower wavelengths, using 670 (29) and 660 nm.(30,31,34). The power of the diode laser also varied between the studies analyzed. Again, the highest powers were found in the LT studies ranging from 1 (17) to 2.5 W (28). One study used 150 mW (29) while three of the six trials using PDT used 100 mW of power (30,31,34). Regarding the application time of the laser irradiation. The two LT studies (17,28) used 30 seconds, one PDT study (29) used 60 seconds of application, while the rest used the laser for 10 seconds (30,31,34). Most of the studies made a single application of the laser (17,29–31,34), while one of the trials (28) made three applications in consecutive days. Two studies (32,33) did not provide in their articles any information on the technical specifications of the diode laser used.

-Risk of bias across studies 

**Figure 2:**
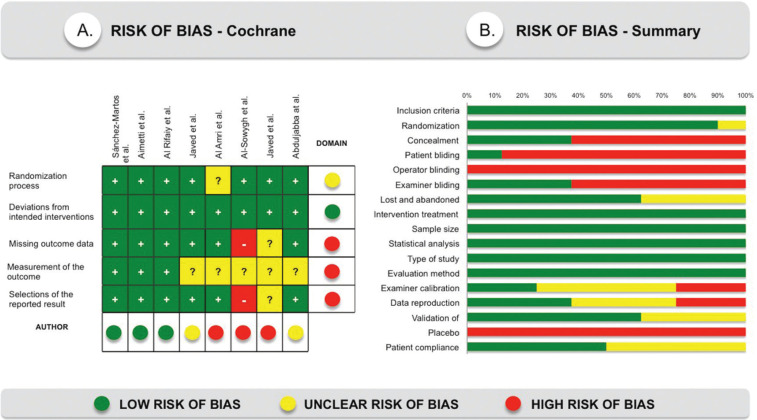
A) Risk of bias according to the Cochrane system. B) Risk of bias summary, review authors’ judgments about each risk of bias item presented as percentages across all included studies.

All included studies were evaluated according to the Cochrane Collaboration tool (20) and Figure 2A, summarizes this analysis. Three of the eight studies were classified as low risk of bias (17,28,29), two had an unclear risk (30,34) and three of the trials had a high risk of bias (31-33). Figure 2B, shows review authors judgments about the other risk of bias items analyzed as percentages across all included studies. All studies specified the inclusion criteria and all, except one (31), also specified the randomization process. Thirty percent of studies performed allocation concealment of the intervention (17,28,29). Only one study blinded the patients (28), none of the trials blinded operators, and 30% blinded examiners (17,28,29). Sixty percent of the studies specified losses and dropouts in the sample and 40% are ambiguous (31-33). Twenty percent of the studies specified the calibration system used, as well as, the intra-examiner agreement (17,28), 40% of the trials only showed the concordance (29,30,32,33) and 20% did not perform a previous examiner calibration (31,34). All the trials specified the type of study, the sample size, the intervention assigned to the test group, the evaluation system and the statistical analyzes.

-Synthesis of the results

Three measures for oral hygiene and peri-implant mucositis (PI, BoP, PPD) had been compared assessing the effect of diode laser. Plaque index (SMD: -1.24; 95% CI: -0.47/-1.53) significantly was reduced after employing diode laser as coadyuvant therapy. For GI (SMD: -0.84; 95% CI: -0.31/-1.53) and probing pocket depth (SMD: -1.36; 95% CI: -0.28/-1.69) not significant statistical differences were found between groups.

## Discussion

This systematic review was focused on evaluating the therapeutic value of diode laser therapy in the treatment of peri-implant mucositis. The current definition is the one proposed by the Workshop of Periodontology in 2017 (6) and only the most recent study (17) of those analyzed in the review follows this definition. However, bleeding on gentle probing is currently considered the key factor for the diagnosis of peri-implant mucositis due to its correlation with inflammation of the peri-implant mucosa at histological level (35). This clinical sign is present in all studies, acting as a common factor between the different descriptions of the pathology.

Considerable heterogeneity was observed in the eight clinical trials with respect to study design, evaluation period, study population, number, gender and age of participants. Discrepancies were also found in sample selection between studies. Two studies used a sample made up only of smokers (30,33) and two others of electronic cigarette smokers (29,32). Smoking has been high lighted as a risk indicator for peri-implant mucositis and can also affect the outcome of treatment (13). Similarly, two studies were carried out on medically compromised patients (31,34). Therefore, having samples that were not representative may lead to the results of these studies not being reproducible in different populations. Conventional non-surgical treatment of peri-implant mucositis and oral hygiene instructions were used as basic therapy in all studies included in the review. However only two most recent trials detailed the procedure (17,28). It is important to note that laser therapy acts as an adjuvant to mechanical debridement and changes in conventional treatment could cause variations in outcomes observed with diode laser therapy, in addition to conditioning the reproducibility of publications.

There was a significant methodological heterogeneity and incomplete information about laser and photosensitizer parameters in the studies included in the review. Variation of any of these parameters could generate totally different clinical effects and, also, affect the obtained results. The latest systematic review concerning the role of diode laser in the treatment of peri-implant mucositis, carried out by Albaker *et al.* in 2012 found the same limitation, specifying that a meta-analysis could not be performed due to the low number of included studies and their variability (22). In the same way, reviews that study the effect of diode laser therapy in the treatment of periodontal diseases obtained similar outcomes, despite the fact that there is more published literature than in the case of peri-implant pathologies. In the review carried out by Atieh *et al.* in 2010 only four RCTs were included and although the meta-analysis was performed subgroup analyses and meta-regression were not attempted given the small number of included studies (9). Therefore, our results regarding the methodology of the trials included in the review are in line with those obtained in past systematic reviews and meta-analyses.

-Plaque index 

There is solid evidence in the scientific literature on the cause-effect relationship between the prevalence of peri-implant mucositis and the accumulation of bacterial biofilm in the peri-implant sulcus (12,13).

**Figure 3:**
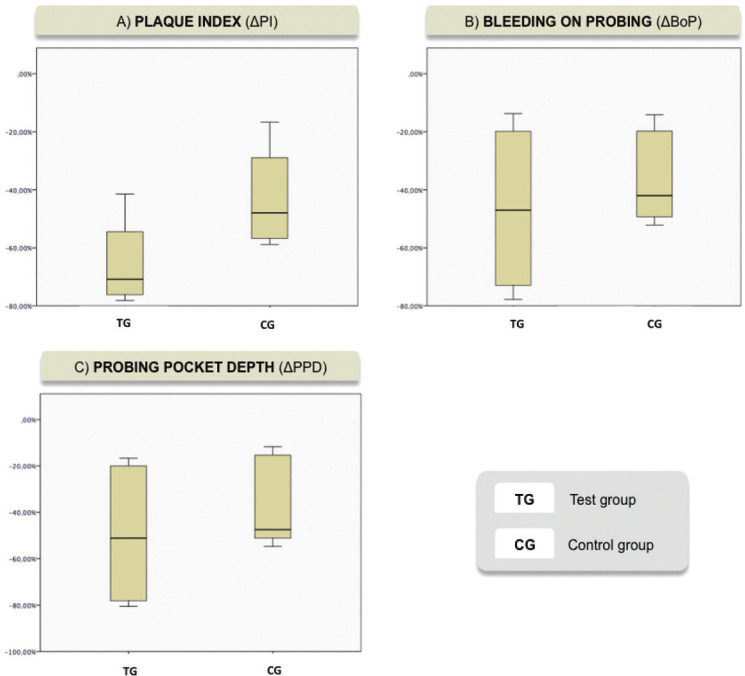
Box plot from meta-analysis showing the reduction in percentage from baseline to 3-month reevaluation A) Reduction of plaque index. B) Reduction of bleeding on probing. C) Reduction of probing pocket depth.

In Figure 3 it can be appreciate that there is a significative greater reduction percentage with respect to plaque index between both groups ( *p*<IC:95% ). In the studies of Aimetti *et al.*, 2019 (28), Sanchez *et al.* 2020 (17), significant differences were not observed in plaque index at 3 months of follow up between both groups (*p*> 0.05). By the other hand, In the studies of Al-Rifaiy *et al.*, 2018 (29), Javed *et al.* 2017 (30), were observed significant differences between groups in plaque index (*p*<0.001) at 3 months follow up. These different outcomes should be carefully interpreted, first of all due to the high risk of bias of the studies (29,30). Second the type of sample may influence the results: sample made only by smokers, have higher plaque index at baseline (29) and, when the intervention were done, the treatment benefits, can be more evident in worse situations.

The effect of the diode laser on the plaque index can be explained by the bactericidal effect of the diode laser favors the conditioning of the peri-implant tissues (21), facilitating the formation of the epithelial seal and therefore reducing the accumulation of biofilm in the peri-implant sulcus, which supposes a direct effect in the main risk factor of peri-implant mucositis (12). It can be explained also by patients behaviour: patients that undergoing to laser group, are not blind in the studies and this, may leads to be more motivated, due to the complexity of this type of treatment (28).

-Bleeding on probing

Bleeding on probing has always been a constant in the definition of peri-implant mucositis (35). Therefore is considered as a clinical sign of reference to evaluate the use of adjuvant treatments in the management of peri-implant pathologies.

In Figure 3, it can be notice that there are not significant differences between both groups with respect to bleeding on probing in the included studies (*p*< IC:95%). In the studies of Aimetti *et al.*, 2019 (28), Al Rifay *et al.*, 2018 (29), Javed *et al.*, 2017 (30), there were not observed significant differences in bleeding on probing score at 3-months follow up between groups. Changes were more pronounced at 1-month follow-up in test group (*p* < 0.01) (28), but at 3-month follow-up both groups showed comparable low residual BoP scores (*p* > 0.05). At 6 months of follow up, Abduljabbar *et al.* didn’t meet statistical differences between groups. These differences maybe can be explained by the fact that the sample is made by smokers and, even if they are vaping smokers, nicotine, other elements and warm temperature, act as a vasoconstriction on mucosa bleed vessels, which results in less bleeding of soft tissues (13). Smoke can alterate the outcomes of non-surgical therapy, with or without adjunctive diode laser therapy. Another fact is that sample size of Aimetti *et al.* (28), is bigger than the others studies, moreover, we need longer follow up times to appreciate differences between groups (15). By the other hand, In the study of Sanchez-Martos *et al.*, 2020 (17) Abduljabbar *et al.*, 2016 (34), there were observed significant differences in bleeding on probing score at 3-months follow up between groups (*p*<0.001). Al-Amri *et al.*, 2016 (31) also observed significant differences in bleeding on probing between both groups at 6 and 12 months follow up (*p*<0.001).

This may be explained by the bactericidal properties of the laser together with biostimulation, allow the reduction of inflammation in the peri-implant tissues (17). However it seems that having toxic habits such as tobacco or systemic diseases that affect the integrity of the peri-implant mucosa, can compromise the restoration of gingival health (13).

-Probing pocket depth

Although the peri-implant mucostits do not show bone loss, inflammatory changes can modify the probing pocket depth (6) and therefore this clinical sign must be taken into account to evaluate the treatments applied to peri-implant mucositis.

In Figure 3 it can be exhibit that there are not significant differences between both groups with respect to probing pocket depth in the included studies. (*p*<IC:95%). In the studies of Sanchez-Martos *et al.* (17) and Aimetti *et al.* (28) and there were not observed statistical differences between groups at 3 months of follow up. In the Aimetti *et al.* (28) study an important factor was the negative effect of a previous history of periodontitis on the treatment outcomes. Patients with chronic periodontitis had statistically significant less reduction in probing pocket depth that those with no history of periodontal disease. This may be due to the transfer of periodontopathogens from the reservoirs of periodontal disease to the implants, conditioning the restoration of the peri-implant mucosa (13). By other hand, Al-Rifaiy *et al.* (29), Javed *et al.* (30) and Abduljabbar *et al.* (34), observed statistical differences in the probing pocket depth between groups at 3 months of follow up (*p*<0.001). In these studies, subjects started with higher probing depths than in other trials. These results seem to indicate, as they showed in the plate index, that the laser seems to be more effective in the most complicated situations. These are due to the fact that its bactericidal action is a critical aid in areas of difficult access such as deep peri-implant pockets, where the effect of mechanical debridement is more limited (21).

The contradictory results obtained in the studies may be due to the fact that although the diode laser allows bacterial decontamination other considerations must be taken into account. Peri-implant tissues exhibit a higher risk of inflammation since they lack one of the main components of the periodontium, the periodontal ligament (15), instead presenting an epithelial seal (35). Therefore, the treatments applied to peri-implant pathologies must not only be able to reduce the clinical signs of the disease, but also show a correlation between these, the microbiological load and the immune response of the host. In our knowledge, currently, there are no clinical trials that address the micorbiological and immunological implications of the use of the diode laser in peri-implant mucositis compared to conventional therapy, however we have promising results when applying this laser therapy in periodontal diseases (36,37). Regarding studies on inflammatory mediators, in the recent *in vitro* study carried out by Chiang *et al.* inflammatory biomarkers, including IL-1b and MMP-8 in the gingival crevicular fluid, were monitored, and the results showed that both cytokine levels were significantly reduced at 4-6 weeks (38). These findings require further investigation, as they could potentially be used in the development of molecular diagnostic utilities and targets for the treatment of peri-implant mucositis (39,40).

This systematic revision and meta-analysis had some limitations, and the results should therefore be interpreted cautiously. The number of included studies was limited because there was a small number of RCTs using diode laser in the treatment of peri-implant mucositis. The heterogeneity in the studies design, also the variety of study population makes it difficult to homogenize and compare the results obtained in the studies. Other variables, apart from those considered in this review, such as: Position, number and implant prosthetic design (single, multiple, screwed or cemented) plays a pivotal role since they can affect the maintenance of good oral higiene and therefore influence the development and resolution of peri-implant mucositis (13). These data are not clearly reflected in the studies and consequently may affect the outcomes.

Within its limits, this systematic review suggests that the use of diode laser, as a coadyuvant in the non-surgical conventional treatment of peri-implant mucositis, is effectiveness in reducing the clinical signs of inflammation caused by peri-implant mucositis, especially in the reduction of peri-implant plaque index. There is a need to carry out more properly designed and performed RCTs that evaluate the effect of diode laser, not only from a clinical perspective, but also microbiological and immunological point of view, to determine what is the true role of diode laser in the treatment of peri-implant pathologies.
